# Differential associations between tongue pressure and handgrip strength with MDS-UPDRS part III motor symptoms in Parkinson’s disease

**DOI:** 10.3389/fneur.2026.1914920

**Published:** 2026-09-17

**Authors:** Haruna Ushimura, Tomoko Yamaji, Kiyoka Seto, Hidetoshi Miyata, Hiromasa Tsujiguchi, Hiroyuki Nakamura, Kazuo Iwasa

**Affiliations:** 1 Faculty of Nursing, Ishikawa Prefectural Nursing University, Kahoku, Japan; 2 Miyata Dental Office, Kanazawa, Japan; 3 Department of Public Health, Ishikawa Prefecture Dental Association, Kanazawa, Japan; 4 Department of Hygiene and Public Health, Faculty of Medicine, Institute of Medical, Pharmaceutical, and Health Sciences, Kanazawa University, Kanazawa, Japan; 5 Department of Health and Medical Sciences, Ishikawa Prefectural Nursing University, Kahoku, Japan

**Keywords:** handgrip strength, movement disorders, Parkinson’s disease, tongue pressure, Unified Parkinson’s Disease Rating Scale

## Abstract

Tongue pressure is a swallowing-related measure that correlates with handgrip strength, an indicator used in the assessment of sarcopenia. However, reduced tongue pressure in Parkinson’s disease (PD) may reflect not only generalized muscle weakness but also PD-related motor control impairment, raising the possibility that tongue pressure and handgrip strength may show different patterns of association with PD motor symptoms. This study examined the patterns of association between tongue pressure and handgrip strength and motor symptoms assessed using Part III of the Movement Disorder Society–sponsored revision of the Unified Parkinson’s Disease Rating Scale (MDS-UPDRS) in patients with PD. A total of 49 individuals with PD aged 70–85 years were enrolled in this cross-sectional observational study. Tongue pressure, handgrip strength, and MDS-UPDRS Part III scores were assessed. Exploratory factor analysis of the MDS-UPDRS Part III items was conducted, followed by multiple regression analyses based on the total score and identified factors. After adjusting for age and sex, tongue pressure (*β* = −0.320, *p* = 0.021) and handgrip strength (*β* = −0.409, *p* = 0.025) were associated with the MDS-UPDRS Part III total score. Three motor symptom factors were identified: axial control and global motor output, limb motor function, and tremor. Tongue pressure was associated with limb motor function involving rigidity and bradykinesia, whereas handgrip strength was associated with axial control and global motor output. These findings suggest that reduced tongue pressure in patients with PD may reflect not only generalized muscle weakness but also specific aspects of PD-related motor dysfunction. However, given the cross-sectional nature of the study, further studies are required to better characterize the relationship between tongue pressure and PD motor symptoms.

## 1 Introduction

Parkinson’s disease (PD) is a progressive neurodegenerative disorder characterized by motor dysfunction resulting from the loss of midbrain dopaminergic neurons. Oropharyngeal dysfunction is frequently observed in patients with PD, and reduced tongue pressure is a commonly reported impairment (1). Tongue pressure, an objective indicator of swallowing function, is considered to reflect tongue muscle strength (2). A reduction in tongue pressure is associated with an increased risk of dysphagia and mortality from aspiration pneumonia (3, 4). Notably, patients with PD reportedly exhibit lower tongue pressure than healthy older adults (5), suggesting that tongue pressure is related to disease-associated motor impairments. Furthermore, tongue pressure has been reported to correlate with disease severity as assessed by the Hoehn and Yahr stage (5, 6). In addition, patients with PD have been reported to show a delayed time to reach maximum tongue pressure and return to baseline compared with healthy controls, suggesting that the motor control required for tongue pressure generation may be impaired in PD (7).

Reduced tongue pressure is commonly associated with sarcopenia (8), a condition characterized by age-related declines in skeletal muscle mass, muscle strength, and physical function. Moreover, decreased handgrip strength, an indicator used in the assessment of sarcopenia, has also been reported to correlate with reduced tongue pressure (9). Notably, the prevalence of sarcopenia among patients with PD ranges from 17 to 62%, which exceeds that observed in the general older adult population (10). Handgrip strength has also been reported to be associated with axial motor symptoms in patients with PD (11, 12). Taken together, reduced tongue pressure in PD may be related to PD-related motor control impairment, including bradykinesia, as well as generalized muscle weakness. However, to the best of our knowledge, no previous study has simultaneously examined the patterns of association of tongue pressure and handgrip strength with PD motor symptoms. If reduced tongue pressure primarily reflects generalized muscle weakness, tongue pressure and handgrip strength would be expected to show similar patterns of association with PD motor symptoms.

The Movement Disorder Society–sponsored revision of the Unified Parkinson’s Disease Rating Scale (MDS-UPDRS) Part III assesses a broad range of PD motor symptoms, including rigidity, bradykinesia, tremor, gait, and postural function, allowing associations with specific motor symptom domains to be examined. Therefore, the present study aimed to examine the patterns of association of tongue pressure and handgrip strength with motor symptoms assessed using the MDS-UPDRS Part III in patients with PD. Clarifying the associations between tongue pressure and PD motor symptoms may help identify patients requiring assessment of tongue and oral motor function, particularly because swallowing impairments in PD may not be fully captured by perceived symptoms (13).

## 2 Materials and methods

### 2.1 Participants

This study was approved by the Ethics Committee of Ishikawa Prefectural Nursing University (No. 2024–82) and adhered to the Declaration of Helsinki. Written informed consent was obtained from all participants before participation.

This cross-sectional study was conducted from January 2025 to June 2025. Participants were recruited from five long-term care facilities and two PD patient support groups. Eligibility criteria included a clinical diagnosis of PD by a neurologist, age between 70 and 85 years, ability to eat independently, and capacity to understand the study procedures and provide informed consent. The age range of 70–85 years was selected to minimize age-related variability and because sex differences in tongue pressure diminish after 70 years of age (14, 15). Exclusion criteria included ongoing treatment for oral disease, a history of dysphagia or choking within the past 6 months, neuromuscular diseases other than PD, and inability to walk attributable to PD. The absence of clinically apparent dysphagia was confirmed based on participant self-report and information provided by facility staff at enrollment. Although cognitive function was not formally assessed, all participants were able to understand and follow the instructions required for the study assessments.

A total of 54 individuals were screened; 4 participants were excluded owing to the age criterion. In addition, one participant exhibited an extreme tongue pressure value (76.9 kPa), exceeding the interquartile range by more than five times, and was therefore excluded as an outlier. Consequently, 49 participants were included in the final analysis (Figure 1).

**Figure 1:**
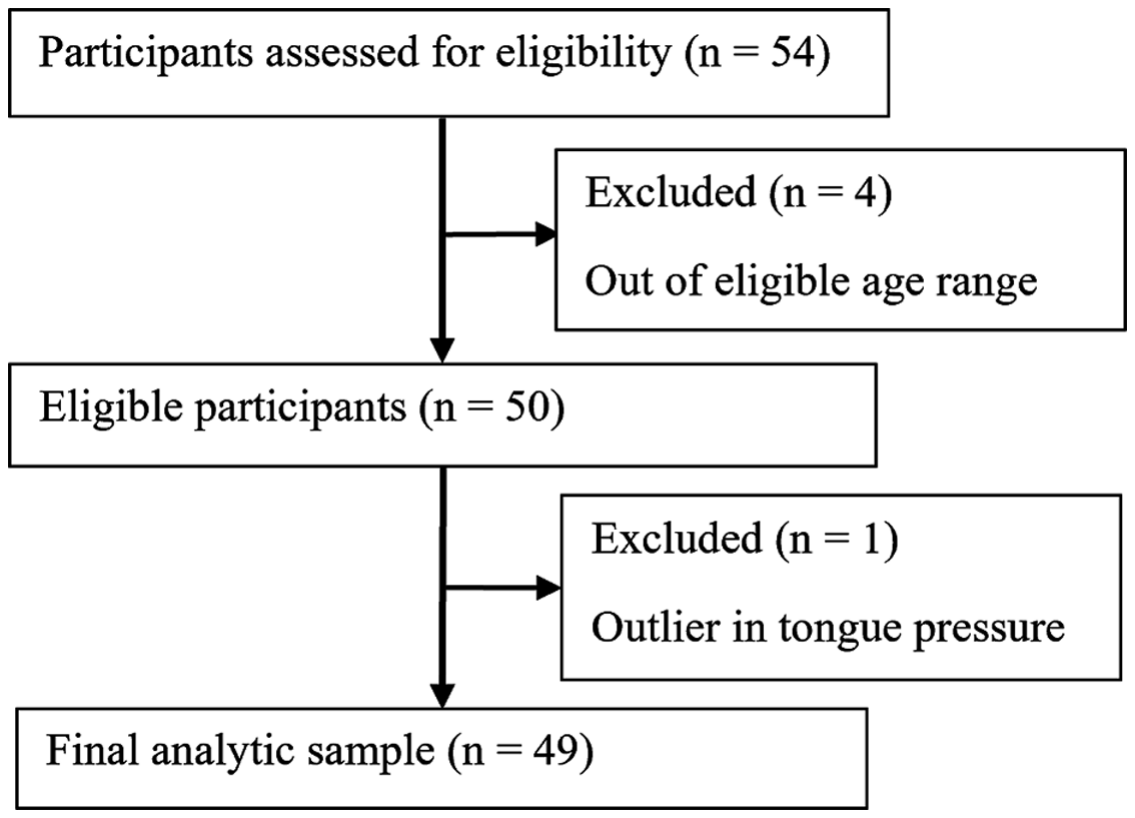
Flow diagram of the participant inclusion and exclusion criteria. Among 54 participants assessed, 4 were excluded owing to age criteria and 1 owing to an outlier in tongue pressure. The final analytic sample comprised 49 participants.

### 2.2 Tongue pressure

Tongue pressure was measured using a TPM-01 device (JMS Co., Ltd., Hiroshima, Japan). The balloon probe was placed against the anterior hard palate, and participants were instructed to press the balloon upward with maximal effort for 7 s. The measurement was performed three times, and the maximum value was used for analysis (7, 16).

### 2.3 Handgrip strength

Handgrip strength was assessed using a Grip-D TKK5401 dynamometer (Takei Scientific Instruments Co., Ltd., Niigata, Japan) with participants in the seated position. Two measurements were obtained from each hand, and the maximum value was used for analysis (17).

### 2.4 Assessment of PD severity (MDS-UPDRS)

Motor symptoms and activities of daily living were assessed using Parts II and III of the MDS-UPDRS (18). The MDS-UPDRS was used with permission from the International Parkinson and Movement Disorder Society. The MDS-UPDRS was administered by a neurologist. For participant safety, assessments were conducted during the ON state whenever possible. Medication state (ON/OFF) was self-reported, and three participants who reported being in the OFF state were included in the analyses because they did not meet any exclusion criteria and were able to safely complete the assessments.

### 2.5 Number of remaining teeth

The number of remaining teeth was assessed directly by a dentist because it has been reported to be associated with tongue pressure (19). When a dentist was not available, assessments were performed using intraoral photographs captured with dentures removed.

### 2.6 Statistical analysis

Participant characteristics were described by sex and compared using the Mann–Whitney U test, chi-square test, or Fisher’s exact test, as appropriate. Data normality was assessed using the Kolmogorov–Smirnov test and the Shapiro–Wilk test. Correlation analyses were conducted using Pearson’s or Spearman’s correlation coefficients, as appropriate. Sex-stratified correlation and item-level analyses of MDS-UPDRS Part III were conducted as exploratory analyses. For the total sample, partial correlation analyses adjusted for sex were additionally performed for the item-level analyses. For MDS-UPDRS Part III items (3.1–3.18), the variables were rank-transformed before the partial correlation analyses.

Exploratory factor analysis was performed using the principal factor method with Promax rotation to classify individual MDS-UPDRS Part III items into motor symptom factors. Items with factor loadings ≥0.40 were considered to demonstrate meaningful loading on a given factor. The number of factors was determined through a comprehensive evaluation of the scree plot, the pattern of decreases in rotated sums of squared loadings, and established clinical classifications of motor symptoms. Factor loadings with absolute values <0.10 were omitted from figures for clarity. Data adequacy for factor analysis was assessed using the Kaiser–Meyer–Olkin measure and Bartlett’s test of sphericity.

Multiple regression analyses were conducted using the extracted factor scores as dependent variables, with tongue pressure, handgrip strength, age, and sex as independent variables. To examine the interpretability of these factor-based findings, additional correlation and regression analyses were performed for individual MDS-UPDRS Part III items. Multiple regression analyses were also performed using the previously reported four-subdomain classification (axial symptoms, rigidity, bradykinesia, and tremor) (20, 21) as the dependent variables. For item-level correlation, partial correlation, and regression analyses of MDS-UPDRS Part III items (3.1–3.18), *p*-values were adjusted for multiple comparisons within each analysis using the Benjamini–Hochberg false discovery rate (FDR) procedure. The MDS-UPDRS Part III total score was not included in the FDR correction.

Statistical analyses were performed using IBM SPSS Statistics version 29 (IBM Ltd., Armonk, NY, United States). Statistical significance was set at *p* < 0.05.

## 3 Results

### 3.1 Participant characteristics

Participant characteristics are summarized in Table 1. The median age was 78.0 years (interquartile range: 75.0–81.0), and 22 participants (44.9%) were men. The median MDS-UPDRS Parts II and III scores were 16.0 (9.0–23.5) and 29.0 (18.0–41.5), respectively. The median tongue pressure was 30.9 (23.7–41.3) kPa for men and 28.6 (23.6–35.3) kPa for women. The median handgrip strength was 25.7 (21.8–31.7) and 18.5 (13.4–21.6) kg for men and women, respectively. Tongue pressure was significantly correlated with handgrip strength in men (*r* = 0.456, *p* = 0.033) and women (*r* = 0.435, *p* = 0.023). No significant correlations were observed between the number of remaining teeth and either tongue pressure or handgrip strength in either sex.

**Table 1:** Characteristics of participants.

Variables		Total (*n* = 49)	Men (*n* = 22)	Women (*n* = 27)	*p*-value
		N (%)/Median (IQR)	n (%)/Median (IQR)	n (%)/Median (IQR)	
Age, years, median (IQR)		78.0 (75.0–81.0)	78.0 (75.0–81.0)	78.0 (76.0–81.0)	0.731^a^
Data collection site, n (%)					0.782^b^
	Nursing home	30 (61.2%)	13 (59.1%)	17 (63.0%)	
	Support group	19 (38.8%)	9 (40.9%)	10 (37.0%)	
Severity of PD					
Hoehn and Yahr, n (%)					0.340^a^
	Yahr2	1 (2.0%)	0 (0.0%)	1 (3.7%)	
	Yahr3	28 (57.1%)	15 (68.2%)	13 (48.1%)	
	Yahr4	20 (40.8%)	7 (31.8%)	13 (48.1%)	
MDS-UPDRS, score, median (IQR)					
	Part II	16.0 (9.0–23.5)	13.0 (8.8–24.0)	16.0 (9.0–24.0)	0.615^a^
	Part III	29.0 (18.0–41.5)	28.0 (18.0–41.3)	33.0 (18.0–43.0)	0.615^a^
Medication state, n (%)					1.000^c^
	On	46 (93.9%)	21 (95.5%)	25 (92.6%)	
	Off	3 (6.1%)	1 (4.5%)	2 (7.4%)	
LEDD, mg, median (IQR)		685.0 (433.2–1062.0)	695.0 (349.4–1019.5)	671.7 (436.4–1155.0)	0.574^a^
Oral condition and function					
Number of remaining teeth, n, median (IQR)		18.0 (10.5–26.0)	22.5 (14.0–26.0)	15 (7.0–26.0)	0.187^a^
Wearing dentures, n (%)		19 (38.8%)	7 (31.8%)	12 (44.4%)	0.367^b^
Tongue pressure, kPa, median (IQR)		29.7 (23.8–38.6)	30.9 (23.7–41.3)	28.6 (23.6–35.3)	0.320^a^
Low tongue pressure, n (%)		9 (18.4%)	4 (18.2%)	5 (18.5%)	1.000^c^
Handgrip strength					
Handgrip strength, kg, median (IQR)		21.3 (15.2–27.2)	25.7 (21.8–31.7)	18.5 (13.4–21.6)	<0.001^a^
Low handgrip strength, n (%)		26 (53.1%)	13 (59.1%)	13 (48.1%)	0.445^b^

Values are presented as median (interquartile range) or n (%). a = Mann–Whitney U test; b = Chi-square test; c = Fisher’s exact test. PD, Parkinson’s disease; MDS-UPDRS, Movement Disorder Society–sponsored revision of the Unified Parkinson’s Disease Rating Scale; LEDD, levodopa equivalent daily dose. Tongue pressure was defined as the maximum value among three measurements. Handgrip strength was defined as the maximum value among four measurements (two measurements for each hand). Low tongue pressure was defined as <20 kPa in men and women. Low handgrip strength was defined as <28 kg in men and <18 kg in women, based on the Asian Working Group for Sarcopenia (AWGS) criteria.

### 3.2 Correlation between tongue pressure or handgrip strength and MDS-UPDRS part III

Tongue pressure was significantly correlated with the MDS-UPDRS Part III total score and several individual items, including rigidity, hand movements, and pronation–supination movements of the hands (Figure 2; Supplementary Table S1). In the sex-stratified analyses, significant correlations between tongue pressure and the MDS-UPDRS Part III total score and individual items were observed only in women (Supplementary Table S1). No significant correlations were observed between tongue pressure and the MDS-UPDRS Part II total score for either sex.

**Figure 2:**
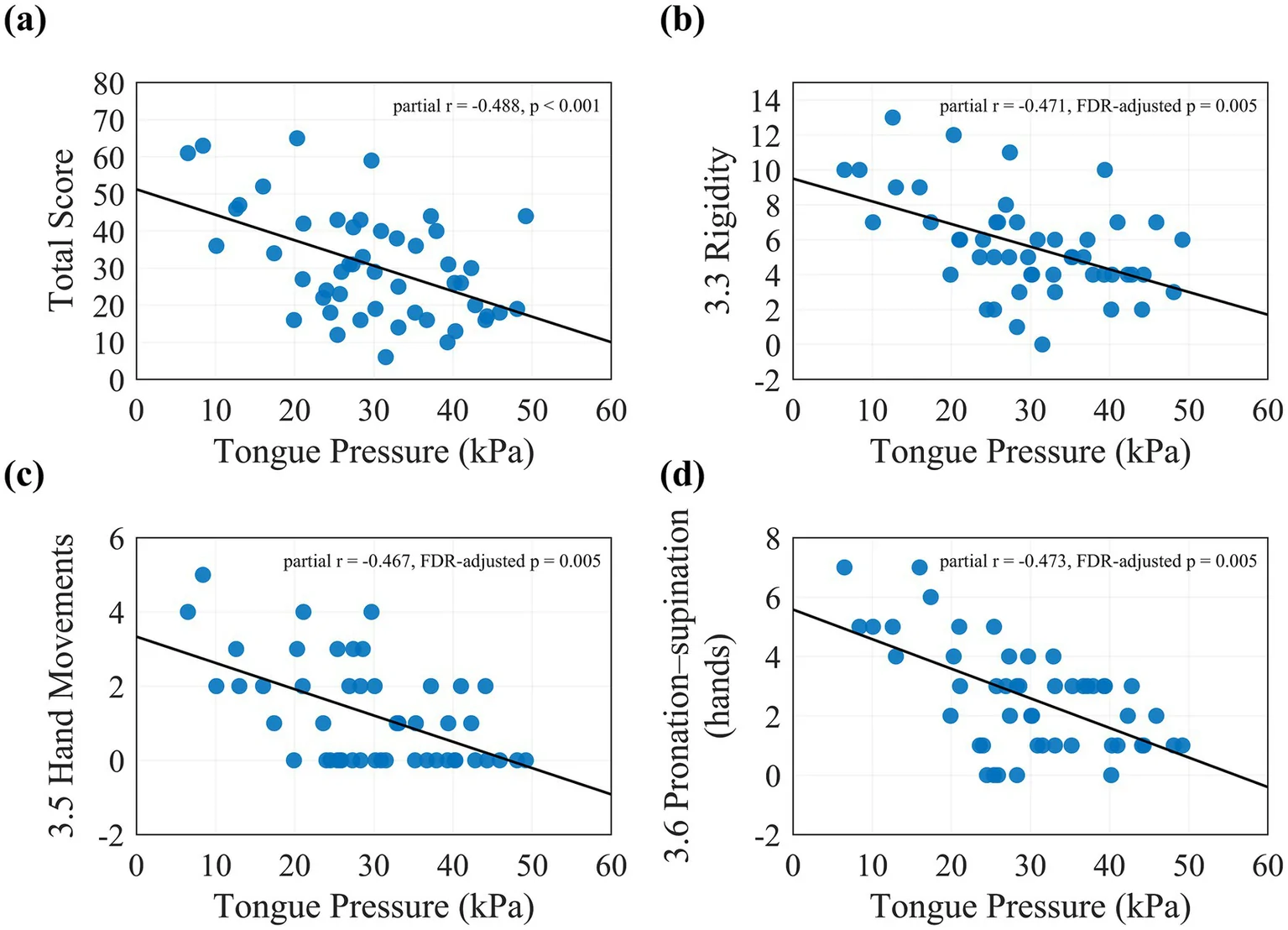
Associations between tongue pressure and MDS-UPDRS Part III scores. Scatterplots depict associations between tongue pressure and MDS-UPDRS Part III scores: **(a)** total score, **(b)** rigidity, **(c)** hand movements, and **(d)** pronation–supination movements of the hands. Lines indicate least-squares regression lines. Reported partial correlation coefficients were adjusted for sex. For the item-level analyses, partial correlations were calculated using rank-transformed variables. *p*-values for MDS-UPDRS Part III items (3.1–3.18) were adjusted for multiple comparisons using the Benjamini–Hochberg false discovery rate (FDR) procedure. The MDS-UPDRS Part III total score was not included in the FDR correction.

Handgrip strength was significantly correlated with the MDS-UPDRS Part III total score in men and women (Supplementary Table S2). Significant correlations were observed between handgrip strength and speech and global spontaneity of movement in each sex. However, a significant correlation between handgrip strength and the MDS-UPDRS Part II total score was observed only in men (ρ = −0.648, *p* = 0.001).

### 3.3 Factor analysis of MDS-UPDRS part III item scores

The dataset met the statistical criteria for factor analysis, with a Kaiser–Meyer–Olkin value of 0.748 and a significant Bartlett’s test of sphericity (χ^2^ = 569.2, df = 153, *p* < 0.001). Exploratory factor analysis yielded a three-factor solution, accounting for 60.6% of the total variance.

Factor 1 was characterized as axial control and global motor output, with high loadings on MDS-UPDRS Part III items 3.1, 3.2, 3.9, 3.10, and 3.12–3.14. Factor 2 was characterized as limb motor function, with high loadings on items 3.3–3.6 and 3.8. Factor 3 was characterized as tremor, with high loadings on items 3.15, 3.17, and 3.18. The detailed factor loadings for each item are presented as a heatmap in Figure 3. The extracted factor structure showed minimal cross-loadings and was largely consistent with established clinical motor features in PD (18, 22).

**Figure 3:**
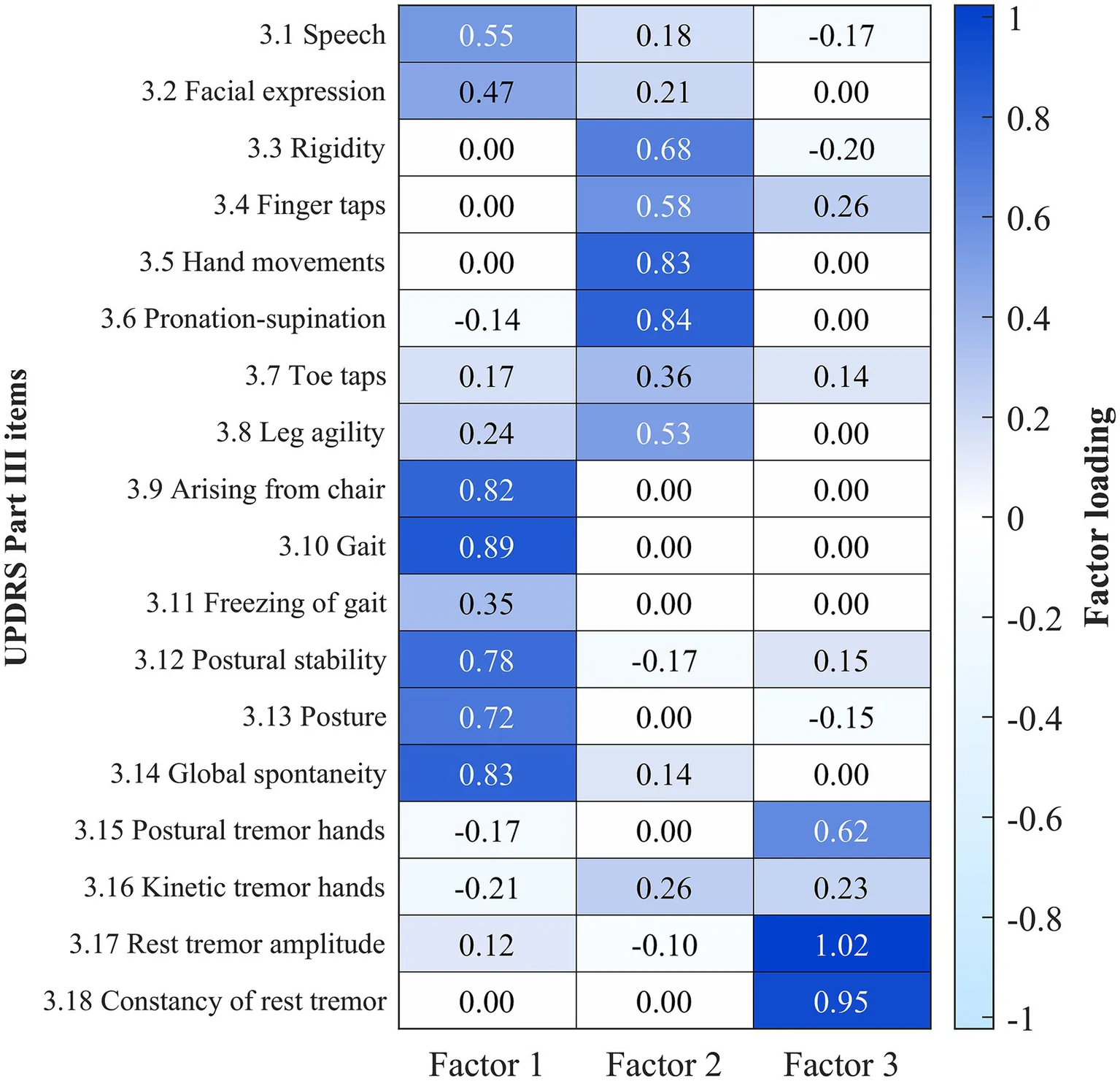
MDS-UPDRS Part III factor loadings (pattern matrix). Exploratory factor analysis was conducted using the principal factor method with Promax rotation. Values represent standardized factor loadings; loadings with absolute values <0.10 were suppressed for clarity. Factor 1 reflected axial control and global motor output. Factor 2 reflected limb motor function with high loadings on bradykinesia/rigidity-related limb items. Factor 3 reflected tremor-related symptoms.

### 3.4 Differing associations of tongue pressure and handgrip strength with motor symptoms

The MDS-UPDRS Part III total score was significantly associated with tongue pressure and handgrip strength after adjusting for age and sex (Table 2).

**Table 2:** Regression results for the MDS-UPDRS Part III total score and factor scores.

Predictor		Model 1 (Univariable model)			Model 2 (Adjusted model)	
	β	B (95% CI)	*p*	β	B (95% CI)	*p*
MDS-UPDRS part III total score						
Tongue pressure (kPa)	−0.496	−0.687 (−1.040 – −0.334)	<0.001	−0.320	−0.443 (−0.816 – −0.071)	0.021
Handgrip strength (kg)	−0.514	−0.990 (−1.475 – −0.506)	<0.001	−0.409	−0.787 (−1.470 – −0.104)	0.025
MDS-UPDRS part III factor 1						
Tongue pressure (kPa)	−0.279	−0.026 (−0.052 – <0.001)	0.052	−0.055	−0.005 (−0.030–0.020)	0.685
Handgrip strength (kg)	−0.529	−0.068 (−0.099 – −0.036)	<0.001	−0.458	−0.058 (−0.104 – −0.013)	0.013
MDS-UPDRS part III factor 2						
Tongue pressure (kPa)	−0.593	−0.053 (−0.074 – −0.032)	<0.001	−0.503	−0.045 (−0.069 – −0.021)	<0.001
Handgrip strength (kg)	−0.401	−0.050 (−0.083 – −0.016)	0.004	−0.229	−0.028 (−0.072–0.016)	0.199
MDS-UPDRS part III factor 3						
Tongue pressure (kPa)	−0.072	−0.007 (−0.035–0.021)	0.621	−0.013	−0.001 (−0.033–0.030)	0.938
Handgrip strength (kg)	−0.104	−0.014 (−0.053–0.025)	0.475	−0.217	−0.029 (−0.087–0.029)	0.319

β = standardized regression coefficient; B = unstandardized regression coefficient; CI = confidence interval. Model 1 represents separate univariable linear regression analyses for tongue pressure and handgrip strength for each outcome. Model 2 represents multivariable linear regression analyses simultaneously including tongue pressure and handgrip strength, with additional adjustment for age and sex. Tongue pressure was defined as the maximum value among three measurements. Handgrip strength was defined as the maximum value among four measurements (two measurements for each hand). Factor 1 reflected axial control and global motor output. Factor 2 reflected limb motor function with high loadings on bradykinesia/rigidity-related limb items. Factor 3 reflected tremor-related symptoms. All variance inflation factors were < 5, indicating no evidence of multicollinearity. Adjusted R^2^ values for Model 2 were as follows: MDS-UPDRS Part III Total Score, 0.335 (*p* < 0.001); Factor 1, 0.324 (*p* < 0.001); Factor 2, 0.336 (*p* < 0.001); Factor 3, 0.001 (*p* = 0.412).

In factor-level analyses, tongue pressure was associated with Factor 2, representing limb motor function, whereas handgrip strength was associated with Factor 1, reflecting axial control and global motor output. No significant associations were observed between the tremor factor and either tongue pressure or handgrip strength.

Sensitivity analyses using mean tongue pressure yielded similar results; in the adjusted model, mean tongue pressure was associated with Factor 2, whereas handgrip strength was associated with Factor 1 (Supplementary Table S3).

In sex-stratified analyses, tongue pressure was significantly associated with the MDS-UPDRS Part III total score and Factor 2 in women (Supplementary Table S4). In contrast, handgrip strength was significantly associated with Factor 1 in both sexes and with the MDS-UPDRS Part III total score only in men.

### 3.5 Clinical interpretation of factor structure

The results of the sensitivity analyses were generally consistent with the factor-level findings. In the multiple regression analyses incorporating the previously reported four-subdomain classification (20, 21), handgrip strength was associated with axial symptoms, whereas tongue pressure was associated with rigidity and bradykinesia (Supplementary Table S5). In the individual-item analyses, tongue pressure was significantly associated with rigidity (3.3), hand movements (3.5), and pronation–supination movements of the hands (3.6) after FDR correction; these were core items contributing to Factor 2 (Supplementary Table S6). In contrast, no individual MDS-UPDRS Part III items were significantly associated with handgrip strength after FDR correction.

## 4 Discussion

In the present study, we investigated the associations of tongue pressure and handgrip strength with motor symptoms in patients with PD. Previous studies have examined the associations of tongue pressure with swallowing-related function and disease severity in patients with PD (23); however, the patterns of association between tongue pressure and PD motor symptoms remain insufficiently understood. We examined the associations of tongue pressure and handgrip strength with different PD motor symptom domains, hypothesizing that they would show similar patterns of association if reduced tongue pressure reflects generalized muscle weakness. Our findings showed that tongue pressure was associated with motor symptoms involving rigidity and upper-limb bradykinesia, whereas handgrip strength was associated with motor symptoms involving axial control and global motor output.

Reduced tongue pressure is associated with sarcopenia (24, 25), and tongue pressure and handgrip strength were also significantly correlated in the present study. However, reduced tongue pressure in PD may not necessarily reflect muscle weakness alone. Patients with PD have been reported to show a longer time to reach maximum tongue pressure and to return to baseline than healthy controls, suggesting temporal abnormalities in tongue pressure generation (7). Furthermore, slower tongue movement has been associated with prolonged oropharyngeal transit time in patients with PD, suggesting that lingual bradykinesia may contribute to impaired swallowing-related tongue function (26). These findings suggest that PD-related abnormalities in the temporal control of tongue movement may also contribute to impaired tongue pressure generation. Basal ganglia circuits affected in PD contribute not only to the control of limb motor symptoms, including bradykinesia and rigidity (27, 28), but also to orofacial motor regulation, including tongue movements (29). Experimental studies in mice have demonstrated that dopaminergic degeneration in the nigrostriatal system is associated with changes in tongue and oropharyngeal motor function (30–32). Our findings extend these previous observations by showing that tongue pressure may be associated with limb motor symptoms involving rigidity and bradykinesia even after accounting for handgrip strength, an indicator of muscle strength.

In contrast, handgrip strength was associated with the MDS-UPDRS Part III total score and Factor 1, which was characterized by items related to axial control and global motor output, including gait (3.10) and global spontaneity of movement (3.14). Handgrip strength is an established indicator of muscle strength and is associated with mobility and gait performance (33). In patients with PD, handgrip strength is reportedly associated with lower-limb muscle strength and axial motor function, including rising from a chair and gait-related items of the UPDRS Part III (12), whereas a recent study found no association between handgrip strength and striatal dopaminergic function (11). Our findings support previous evidence that handgrip strength is associated with axial motor function in patients with PD.

In the present study, sex-stratified exploratory analyses revealed significant correlations between tongue pressure and MDS-UPDRS Part III items exclusively in women. Sex differences have been reported in PD across multiple neuroanatomical domains, including patterns of substantia nigra degeneration and thalamic atrophy (34, 35), cortical thinning, and structural connectivity (36–39). Notably, these differences may contribute to sex-specific associations between tongue pressure and motor symptoms. However, as our study was not designed or statistically powered to evaluate sex differences, these findings should be interpreted cautiously.

Although the factor structure identified in this study and its clinical interpretation are hypothesis-generating rather than definitive, the observed associations of tongue pressure and handgrip strength suggest that tongue pressure may capture aspects of motor dysfunction in PD. Therefore, future studies incorporating larger sample sizes, neuroimaging analyses, and longitudinal designs are required to further elucidate these associations.

The present study has certain limitations. First, the sample size was modest, limiting the statistical power of the multivariable analyses and our ability to fully adjust for multiple potential confounders, such as cognitive function, disease duration, and the number of remaining teeth, which may influence motor symptoms or oral motor function. In particular, previous studies have reported associations of cognitive function with tongue pressure (40) and handgrip strength (41), and disease duration was based on self-report because medical records were unavailable. Future studies should incorporate standardized cognitive assessments and reliable data on disease duration. In addition, although handgrip strength was used as an indicator of muscle strength, sarcopenia was not formally assessed; therefore, findings related to sarcopenia should be interpreted with caution. The absence of clinically apparent dysphagia was determined based on participant self-report and information provided by facility staff, and no instrumental swallowing assessment was performed. Given the reported discrepancy between subjective and objective assessments of dysphagia in PD (13), unrecognized dysphagia may have been present; therefore, the present findings should not be interpreted as reflecting swallowing function as a whole. Second, three participants in the OFF state were included, and residual confounding related to dopaminergic treatment effects cannot be excluded. Third, the cross-sectional design precludes causal inference, and the observed associations may partly reflect unmeasured confounding. Fourth, the study sample was restricted to individuals aged 70–85 years and ambulatory patients with Hoehn and Yahr stages 2–4, limiting the generalizability of the findings, particularly to patients in their 60s and those with Hoehn and Yahr stage 5, as the observed associations may vary with age and disease severity. Finally, the factor structure should be interpreted as exploratory because of the relatively small sample size compared with the number of MDS-UPDRS Part III items. Although the factor-level association between handgrip strength and axial symptoms was also observed using the previously reported four-subdomain classification, no individual MDS-UPDRS Part III items remained significantly associated with handgrip strength after FDR correction. Therefore, the associations between handgrip strength and individual items should be interpreted cautiously. The small sample size and skewed distribution of MDS-UPDRS Part III scores among men also limited the sex-stratified analyses and precluded formal sex × tongue pressure interaction analyses; therefore, the sex-specific findings should be interpreted with caution.

## 5 Conclusion

In this study, we examined the patterns of association of tongue pressure and handgrip strength with motor symptoms in patients with PD. Using exploratory factor analysis followed by regression analyses, our results suggested that tongue pressure and handgrip strength were associated with different motor symptom domains of the MDS-UPDRS Part III. Overall, this study suggests that investigating tongue motor function and motor symptoms in PD may contribute to a better understanding of dysphagia in this population and may help inform future dysphagia assessment and rehabilitation strategies. Larger studies incorporating neuroimaging analyses and longitudinal designs are warranted to further clarify these associations.

## Data Availability

The raw data supporting the conclusions of this article will be made available by the authors, without undue reservation.
