# Supplementary material for: Differential associations between tongue pressure and handgrip strength with MDS-UPDRS part III motor symptoms in Parkinson’s disease
Source: Front Neurol. 2026 Sep 17;17:1914920. doi: 10.3389/fneur.2026.1914920 (PMC13626904; doi:10.3389/fneur.2026.1914920)
Supplement: Supplementary file 1 [file Table_1.docx]

Supplementary Material

# 1 Supplementary Tables

**Supplementary Table 1.** Associations between tongue pressure and MDS-UPDRS Part III scores.

|  | Total (N = 49) | | |  | Men (n = 22) | | |  | Women (n = 27) | | |
| --- | --- | --- | --- | --- | --- | --- | --- | --- | --- | --- | --- |
| Variables | Partial correlation coefficient | p-value | FDR-adjusted  p-value |  | Correlation Coefficient | p-value | FDR-adjusted  p-value |  | Correlation Coefficient | p-value | FDR-adjusted  p-value |
| Total score | **-0.488** | **0.000** | **—** |  | -0.377 | 0.084 | **—** |  | **-0.578** | **0.002** | **—** |
| 3.1 Speech | **-0.358** | **0.012** | 0.056 |  | -0.295 | 0.182 | 0.756 |  | **-0.388** | **0.045** | 0.130 |
| 3.2 Facial expression | **-0.326** | **0.024** | 0.061 |  | -0.164 | 0.467 | 0.756 |  | **-0.449** | **0.019** | 0.080 |
| 3.3 Rigidity | **-0.471** | **0.001** | **0.005** |  | -0.266 | 0.231 | 0.756 |  | **-0.650** | **<0.001** | **<0.001** |
| 3.4 Finger tapping | -0.251 | 0.085 | 0.162 |  | -0.138 | 0.539 | 0.756 |  | -0.380 | 0.051 | 0.130 |
| 3.5 Hand movements | **-0.467** | **0.001** | **0.005** |  | -0.353 | 0.107 | 0.756 |  | **-0.578** | **0.002** | **0.010** |
| 3.6 Pronation–supination movements of hands | **-0.473** | **0.001** | **0.005** |  | -0.415 | 0.055 | 0.756 |  | **-0.504** | **0.007** | **0.040** |
| 3.7 Toe tapping | **-0.337** | **0.019** | 0.061 |  | -0.247 | 0.268 | 0.756 |  | -0.369 | 0.058 | 0.130 |
| 3.8 Leg agility | **-0.331** | **0.022** | 0.061 |  | -0.220 | 0.326 | 0.756 |  | **-0.423** | **0.028** | 0.100 |
| 3.9 Arising from chair | -0.247 | 0.090 | 0.162 |  | -0.252 | 0.257 | 0.756 |  | -0.214 | 0.283 | 0.420 |
| 3.10 Gait | -0.222 | 0.129 | 0.211 |  | -0.184 | 0.411 | 0.756 |  | -0.235 | 0.238 | 0.390 |
| 3.11 Freezing of gait | 0.005 | 0.970 | 0.970 |  | -0.023 | 0.920 | 0.920 |  | 0.023 | 0.910 | 0.910 |
| 3.12 Postural stability | -0.093 | 0.529 | 0.722 |  | -0.135 | 0.548 | 0.756 |  | -0.065 | 0.747 | 0.790 |
| 3.13 Posture | 0.074 | 0.618 | 0.742 |  | -0.038 | 0.865 | 0.920 |  | 0.184 | 0.359 | 0.500 |
| 3.14 Global spontaneity of movement | -0.266 | 0.068 | 0.152 |  | -0.201 | 0.371 | 0.756 |  | -0.330 | 0.093 | 0.190 |
| 3.15 Postural tremor of the hands | -0.041 | 0.783 | 0.836 |  | 0.122 | 0.588 | 0.756 |  | -0.149 | 0.460 | 0.550 |
| 3.16 Kinetic tremor of the hands | -0.086 | 0.562 | 0.722 |  | -0.085 | 0.707 | 0.848 |  | -0.075 | 0.709 | 0.790 |
| 3.17 Rest tremor amplitude | -0.039 | 0.790 | 0.836 |  | 0.122 | 0.588 | 0.756 |  | -0.165 | 0.410 | 0.530 |
| 3.18 Constancy of rest tremor | -0.148 | 0.315 | 0.473 |  | -0.034 | 0.879 | 0.920 |  | -0.247 | 0.214 | 0.390 |
| For the total sample, partial correlation analyses adjusted for sex were performed. Pearson’s partial correlation coefficient was used for the MDS-UPDRS Part III total score.  For Items 3.1–3.18, partial correlations between ranked variables adjusted for sex were performed. For the sex-stratified analyses, Pearson’s correlation coefficient was used for the total score, and Spearman’s rank correlation coefficients were used for Items 3.1–3.18.  p-values for Items 3.1–3.18 were adjusted for multiple comparisons using the Benjamini–Hochberg false discovery rate (FDR) procedure. The MDS-UPDRS Part III total score was not included in the FDR correction. Tongue pressure was defined as the maximum value among three measurements. Bold values indicate p < 0.05 or FDR-adjusted p < 0.05. MDS-UPDRS = Movement Disorder Society–sponsored revision of the Unified Parkinson’s Disease Rating Scale; FDR = false discovery rate. | | | | | | | | | | | |

**Supplementary Table 2.** Associations between handgrip strength and MDS-UPDRS Part III scores.

|  | Total (N = 49) | | |  | Men (n = 22) | | |  | Women (n = 27) | | |
| --- | --- | --- | --- | --- | --- | --- | --- | --- | --- | --- | --- |
| Variables | Partial correlation coefficient | p-value | FDR-adjusted  p-value |  | Correlation Coefficient | p-value | FDR-adjusted  p-value |  | Correlation Coefficient | p-value | FDR-adjusted  p-value |
| Total score | **-0.543** | **<0.001** | **—** |  | **-0.626** | **0.002** | **—** |  | **-0.489** | **0.010** | **—** |
| 3.1 Speech | **-0.626** | **<0.001** | **<0.001** |  | **-0.690** | **<0.001** | **0.007** |  | **-0.616** | **0.001** | **0.010** |
| 3.2 Facial expression | **-0.524** | **<0.001** | **0.001** |  | **-0.518** | **0.014** | 0.058 |  | **-0.556** | **0.003** | **0.020** |
| 3.3 Rigidity | **-0.356** | **0.013** | **0.029** |  | -0.327 | 0.138 | 0.276 |  | -0.331 | 0.092 | 0.180 |
| 3.4 Finger tapping | **-0.294** | **0.043** | 0.077 |  | -0.097 | 0.668 | 0.801 |  | **-0.383** | **0.049** | 0.130 |
| 3.5 Hand movements | **-0.295** | **0.042** | 0.077 |  | -0.218 | 0.331 | 0.496 |  | -0.291 | 0.140 | 0.250 |
| 3.6 Pronation–supination movements of hands | -0.231 | 0.114 | 0.164 |  | -0.160 | 0.478 | 0.615 |  | -0.222 | 0.266 | 0.400 |
| 3.7 Toe tapping | **-0.286** | **0.049** | 0.080 |  | -0.298 | 0.179 | 0.322 |  | -0.272 | 0.170 | 0.280 |
| 3.8 Leg agility | **-0.447** | **0.001** | **0.004** |  | -0.341 | 0.120 | 0.271 |  | **-0.444** | **0.020** | 0.060 |
| 3.9 Arising from chair | **-0.507** | **<0.001** | **0.001** |  | **-0.504** | **0.017** | 0.058 |  | **-0.480** | **0.011** | **0.040** |
| 3.10 Gait | **-0.498** | **<0.001** | **0.001** |  | **-0.494** | **0.019** | 0.058 |  | **-0.506** | **0.007** | **0.030** |
| 3.11 Freezing of gait | -0.078 | 0.599 | 0.718 |  | -0.190 | 0.396 | 0.549 |  | 0.118 | 0.557 | 0.670 |
| 3.12 Postural stability | **-0.439** | **0.002** | **0.005** |  | **-0.630** | **0.002** | **0.015** |  | -0.334 | 0.088 | 0.180 |
| 3.13 Posture | -0.228 | 0.118 | 0.164 |  | -0.439 | 0.041 | 0.105 |  | -0.128 | 0.525 | 0.670 |
| 3.14 Global spontaneity of movement | **-0.585** | **<0.001** | **<0.001** |  | **-0.578** | **0.005** | **0.029** |  | **-0.615** | **0.001** | **0.010** |
| 3.15 Postural tremor of the hands | 0.003 | 0.983 | 0.983 |  | 0.000 | 1.000 | 1.000 |  | 0.037 | 0.854 | 0.850 |
| 3.16 Kinetic tremor of the hands | 0.201 | 0.172 | 0.221 |  | 0.239 | 0.284 | 0.464 |  | 0.176 | 0.379 | 0.520 |
| 3.17 Rest tremor amplitude | -0.034 | 0.816 | 0.864 |  | 0.000 | 1.000 | 1.000 |  | -0.079 | 0.697 | 0.780 |
| 3.18 Constancy of rest tremor | -0.039 | 0.792 | 0.864 |  | -0.055 | 0.807 | 0.907 |  | -0.057 | 0.779 | 0.820 |
| For the total sample, partial correlation analyses adjusted for sex were performed. Pearson’s partial correlation coefficient was used for the MDS-UPDRS Part III total score.  For Items 3.1–3.18, partial correlations between ranked variables adjusted for sex were performed. For the sex-stratified analyses, Pearson’s correlation coefficient was used for the total score, and Spearman’s rank correlation coefficients were used for Items 3.1–3.18.  p-values for Items 3.1–3.18 were adjusted for multiple comparisons using the Benjamini–Hochberg false discovery rate (FDR) procedure. The MDS-UPDRS Part III total score was not included in the FDR correction. Handgrip strength was defined as the maximum value among four measurements (two measurements for each hand).  Bold values indicate p < 0.05 or FDR-adjusted p < 0.05. MDS-UPDRS = Movement Disorder Society–sponsored revision of the Unified Parkinson’s Disease Rating Scale; FDR = false discovery rate. | | | | | | | | | | | |

**Supplementary Table 3.** Sensitivity analysis of multiple regression models for MDS-UPDRS Part III total and factor scores using mean tongue pressure.

|  | MDS-UPDRS Part Ⅲ　Total　Score | | | | | | |
| --- | --- | --- | --- | --- | --- | --- | --- |
|  | Model 1 (Univariable model) | | |  | Model 2 (Adjusted model) | | |
| Predictor | β | B (95%CI) | p |  | β | B (95%CI) | p |
| Mean tongue pressure (kPa) | -0.550 | -0.816 (-1.180 – -0.452) | < 0.001 |  | -0.383 | -0.568 (-0.969 – -0.167) | 0.007 |
| Handgrip strength (kg) | -0.514 | -0.990 (-1.475 – -0.506) | < 0.001 |  | -0.342 | -0.658 (-1.348 – 0.033) | 0.061 |
|  | MDS-UPDRS Part Ⅲ　Factor 1 | | | | | | |
|  | Model 1 (Univariable model) | | |  | Model 2 (Adjusted model) | | |
| Predictor | β | B (95%CI) | p |  | β | B (95%CI) | p |
| Mean tongue pressure (kPa) | -0.331 | -0.033 (-0.060 – -0.005) | 0.020 |  | -0.107 | -0.011 (-0.038 – 0.017) | 0.441 |
| Handgrip strength (kg) | -0.529 | -0.068 (-0.099 – -0.036) | < 0.001 |  | -0.419 | -0.054 (-0.101 – -0.006) | 0.027 |
|  | MDS-UPDRS Part Ⅲ　Factor 2 | | | | | | |
|  | Model 1 (Univariable model) | | |  | Model 2 (Adjusted model) | | |
| Predictor | β | B (95%CI) | p |  | β | B (95%CI) | p |
| Mean tongue pressure (kPa) | -0.639 | -0.061 (-0.083 – -0.040) | < 0.001 |  | -0.567 | -0.054 (-0.080 – -0.029) | < 0.001 |
| Handgrip strength (kg) | -0.401 | -0.050 (-0.083 – -0.016) | 0.004 |  | -0.147 | -0.018 (-0.062 – 0.026) | 0.410 |
|  | MDS-UPDRS Part Ⅲ　Factor 3 | | | | | | |
|  | Model 1 (Univariable model) | | |  | Model 2 (Adjusted model) | | |
| Predictor | β | B (95%CI) | p |  | β | B (95%CI) | p |
| Mean tongue pressure (kPa) | -0.053 | -0.005 (-0.035 – 0.025) | 0.718 |  | 0.011 | 0.001 (-0.034 – 0.036) | 0.950 |
| Handgrip strength (kg) | -0.104 | -0.014 (-0.053 – 0.025) | 0.475 |  | -0.232 | -0.031 (-0.091 – 0.029) | 0.305 |
| Sensitivity analyses were performed using the mean tongue pressure obtained from three measurements instead of the maximum tongue pressure used in the primary analyses. Handgrip strength was defined as the maximum value among four measurements (two measurements for each hand).  β = standardized regression coefficient; B = unstandardized regression coefficient; CI = confidence interval. Model 1 represents separate univariable linear regression analyses for tongue pressure and handgrip strength for each outcome. Model 2 represents multivariable linear regression analyses simultaneously including tongue pressure and handgrip strength, with additional adjustment for age and sex. Factor 1 reflected axial control and global motor output.  Factor 2 reflected limb motor function with high loadings on bradykinesia/rigidity-related limb items.  Factor 3 reflected tremor-related symptoms. All variance inflation factors were < 5, indicating no evidence of multicollinearity. Adjusted R² values for Model 2 were as follows: MDS-UPDRS Part III total Score, 0.365 (p < 0.001); Factor 1, 0.330 (p < 0.001); Factor 2, 0.380 (p < 0.001); Factor 3, 0.001 (p = 0.412). | | | | | | | |

**Supplementary Table 4.** Regression analyses of the sex-stratified associations of tongue pressure and handgrip strength with the MDS-UPDRS Part III total score and factor scores.

| Predictor | Sex | Tongue pressure β (p) | Handgrip strength β (p) |
| --- | --- | --- | --- |
| MDS-UPDRS Part Ⅲ Total　Score | Men | -0.116 (0.568) | **-0.573 (0.010)** |
|  | Women | **-0.450 (0.017)** | -0.293 (0.107) |
|  |  |  |  |
| Factor 1 | Men | -0.045 (0.834) | **-0.590 (0.012)** |
|  | Women | -0.087 (0.654) | **-0.492 (0.017)** |
|  |  |  |  |
| Factor 2 | Men | -0.395 (0.072) | -0.299 (0.166) |
|  | Women | **-0.572 (0.003)** | -0.165 (0.341) |
|  |  |  |  |
| Factor 3 | Men | -0.141 (0.589) | -0.033 (0.897) |
|  | Women | -0.182 (0.420) | -0.043 (0.849) |
| Values are standardized regression coefficients (β) with p-values in parentheses. Multivariable linear regression analyses were performed separately by sex, simultaneously including tongue pressure and handgrip strength as independent variables. Tongue pressure was defined as the maximum value among three measurements. Handgrip strength was defined as the maximum value among four measurements (two measurements for each hand).  Bold values indicate p < 0.05. MDS-UPDRS = Movement Disorder Society–sponsored revision of the Unified Parkinson’s Disease Rating Scale. | | | |

**Supplementary Table 5**. Regression analysis results for the previously reported four-subdomain classification of the MDS-UPDRS Part III.

|  | Axial | | | | | | |
| --- | --- | --- | --- | --- | --- | --- | --- |
|  | Model 1 (Unadjusted) | | |  | Model 2 (Adjusted) | | |
| Predictor | β | B (95%CI) | p-value |  | β | B (95%CI) | p-value |
| Tongue pressure (kPa) | -0.231 | -0.122 (-0.272 – 0.029) | 0.111 |  | -0.008 | -0.004 (-0.146 – 0.137) | 0.950 |
| Handgrip strength　(kg) | -0.526 | -0.386(-0.569 – -0.203) | **< 0.001** |  | -0.413 | -0.303 (-0.562 – -0.044) | **0.023** |
|  | Rigidity | | | | | | |
|  | Model 1 (Unadjusted) | | |  | Model 2 (Adjusted) | | |
| Predictor | β | B (95%CI) | p-value |  | β | B (95%CI) | p-value |
| Tongue pressure (kPa) | -0.488 | -0.130 (-0.198 – -0.062) | **< 0.001** |  | -0.437 | -0.116 (-0.192 – -0.041) | **0.003** |
| Handgrip strength　(kg) | -0.280 | -0.104 (-0.208 – 0.001) | 0.051 |  | -0.137 | -0.051 (-0.189 – 0.088) | 0.465 |
|  | Bradykinesia | | | | | | |
|  | Model 1 (Unadjusted) | | |  | Model 2 (Adjusted) | | |
| Predictor | β | B (95%CI) | p-value |  | β | B (95%CI) | p-value |
| Tongue pressure (kPa) | -0.511 | -0.415 (-0.620 – -0.210) | **< 0.001** |  | -0.367 | -0.298 (-0.522 – -0.073) | **0.011** |
| Handgrip strength　(kg) | -0.472 | -0.532 (-0.824 – -0.240) | **0.001** |  | -0.334 | -0.377 (-0.789 – 0.035) | 0.072 |
|  | Tremor | | | | | | |
|  | Model 1 (Unadjusted) | | |  | Model 2 (Adjusted) | | |
| Predictor | β | B (95%CI) | p-value |  | β | B (95%CI) | p-value |
| Tongue pressure (kPa) | -0.068 | -0.023 (-0.120 – 0.074) | 0.641 |  | -0.039 | -0.013 (-0.123 – 0.097) | 0.813 |
| Handgrip strength　(kg) | -0.056 | -0.026 (-0.161 – 0.109) | 0.702 |  | -0.135 | -0.062 (-0.264 – 0.140) | 0.537 |
| n = 49. Bold values indicate p < 0.05. MDS-UPDRS = Movement Disorder Society–sponsored revision of the Unified Parkinson’s Disease Rating Scale. Axial = MDS-UPDRS items 3.1, 3.9–3.13; Rigidity = 3.3; Bradykinesia = 3.2, 3.4–3.9, 3.14; Tremor = 3.15–3.18. Model 1 represents univariable linear regression analyses for each motor subdomain of the MDS-UPDRS Part III. Model 2 represents multivariable linear regression analyses simultaneously including tongue pressure and handgrip strength, with additional adjustment for age and sex. Tongue pressure was defined as the maximum value among three measurements. Handgrip strength was defined as the maximum value among four measurements (two measurements for each hand).  β indicates standardized regression coefficients, and B indicates unstandardized regression coefficients. All variance inflation factors were < 5, indicating no evidence of multicollinearity. Adjusted R² values for Model 2 were as follows: Axial, 0.341 (p < 0.001); Rigidity, 0.264 (p = 0.001); Bradykinesia, 0.296 (p < 0.001); Tremor, -0.014 (p = 0.513). | | | | | | | |

**Supplementary Table 6**. Regression analyses of the Associations of MDS-UPDRS Part III items with tongue pressure and handgrip strength.

|  | Tongue Pressure | | | | | | |  | Handgrip Strength | | | | | | |
| --- | --- | --- | --- | --- | --- | --- | --- | --- | --- | --- | --- | --- | --- | --- | --- |
|  | Model 1 | | |  | Model 2 | | |  | Model 1 | | |  | Model 2 | | |
| Predictor | β | p-value | FDR-adjusted  p-value |  | β | p-value | FDR-adjusted  p-value |  | β | p-value | FDR-adjusted  p-value |  | β | p-value | FDR-adjusted  p-value |
| 3.1 Speech | -0.335 | 0.019 | 0.056 |  | -0.123 | 0.373 | 0.643 |  | -0.452 | 0.001 | 0.004 |  | **-0.570** | **0.003** | 0.051 |
| 3.2 Facial expression | -0.381 | 0.007 | 0.031 |  | -0.228 | 0.125 | 0.483 |  | -0.357 | 0.012 | 0.031 |  | **-0.476** | **0.017** | 0.080 |
| 3.3 Rigidity | -0.488 | <0.001 | 0.002 |  | **-0.437** | **0.003** | **0.020** |  | -0.280 | 0.051 | 0.102 |  | -0.137 | 0.465 | 0.557 |
| 3.4 Finger tapping | -0.313 | 0.028 | 0.073 |  | -0.245 | 0.134 | 0.483 |  | -0.266 | 0.065 | 0.109 |  | -0.145 | 0.497 | 0.559 |
| 3.5 Hand movements | -0.539 | <0.001 | 0.001 |  | **-0.504** | **0.001** | **0.008** |  | -0.257 | 0.075 | 0.109 |  | -0.153 | 0.416 | 0.535 |
| 3.6 Pronation–supination movements of hands | -0.581 | <0.001 | <0.001 |  | **-0.604** | **<0.001** | **0.001** |  | -0.288 | 0.045 | 0.101 |  | 0.177 | 0.320 | 0.444 |
| 3.7 Toe tapping | -0.194 | 0.181 | 0.296 |  | -0.068 | 0.676 | 0.850 |  | -0.261 | 0.070 | 0.109 |  | -0.374 | 0.087 | 0.196 |
| 3.8 Leg agility | -0.286 | 0.046 | 0.092 |  | -0.135 | 0.393 | 0.643 |  | -0.359 | 0.011 | 0.031 |  | -0.402 | 0.058 | 0.196 |
| 3.9 Arising from chair | -0.298 | 0.037 | 0.084 |  | -0.143 | 0.261 | 0.588 |  | -0.479 | <0.001 | 0.002 |  | -0.179 | 0.286 | 0.429 |
| 3.10 Gait | -0.262 | 0.069 | 0.125 |  | -0.057 | 0.676 | 0.850 |  | -0.540 | <0.001 | 0.001 |  | -0.331 | 0.071 | 0.196 |
| 3.11 Freezing of gait | 0.021 | 0.886 | 0.928 |  | 0.046 | 0.780 | 0.850 |  | -0.015 | 0.920 | 0.920 |  | -0.065 | 0.766 | 0.766 |
| 3.12 Postural stability | -0.178 | 0.222 | 0.333 |  | 0.056 | 0.692 | 0.850 |  | -0.519 | 0.000 | 0.001 |  | **-0.454** | **0.018** | 0.080 |
| 3.13 Posture | 0.046 | 0.753 | 0.903 |  | 0.186 | 0.249 | 0.588 |  | -0.253 | 0.079 | 0.109 |  | -0.249 | 0.241 | 0.408 |
| 3.14 Global spontaneity of movement | -0.338 | 0.017 | 0.056 |  | -0.124 | 0.370 | 0.643 |  | -0.497 | 0.000 | 0.002 |  | -0.507 | 0.007 | 0.067 |
| 3.15 Postural tremor of the hands | 0.013 | 0.928 | 0.928 |  | 0.025 | 0.882 | 0.882 |  | 0.017 | 0.905 | 0.920 |  | -0.105 | 0.631 | 0.668 |
| 3.16 Kinetic tremor of the hands | -0.086 | 0.555 | 0.713 |  | -0.187 | 0.258 | 0.588 |  | 0.054 | 0.715 | 0.804 |  | 0.379 | 0.085 | 0.196 |
| 3.17 Rest tremor amplitude | -0.034 | 0.816 | 0.917 |  | 0.041 | 0.803 | 0.850 |  | -0.096 | 0.511 | 0.643 |  | -0.284 | 0.190 | 0.381 |
| 3.18 Constancy of rest tremor | -0.105 | 0.474 | 0.657 |  | -0.049 | 0.759 | 0.850 |  | -0.091 | 0.536 | 0.643 |  | -0.247 | 0.250 | 0.408 |
| Values are standardized regression coefficients (β). Model 1 represents univariable linear regression analyses for each MDS-UPDRS Part III item. Model 2 represents multivariable linear regression analyses simultaneously including tongue pressure and handgrip strength, with additional adjustment for age and sex. p-values for the 18 MDS-UPDRS Part III items were adjusted for multiple comparisons within each model and predictor using the Benjamini–Hochberg false discovery rate (FDR) procedure. Tongue pressure was defined as the maximum value among three measurements. Handgrip strength was defined as the maximum value among four measurements (two measurements for each hand).  Bold values indicate Model 2 associations with p < 0.05 and/or FDR-adjusted p < 0.05. MDS-UPDRS = Movement Disorder Society–sponsored revision of the Unified Parkinson’s Disease Rating Scale; FDR = false discovery rate. | | | | | | | | | | | | | | | |
